# Prevalence and contextual factors associated with compassion fatigue among nurses in northern Uganda

**DOI:** 10.1371/journal.pone.0257833

**Published:** 2021-09-27

**Authors:** Amir Kabunga, Lucas Goodgame Anyayo, Ponsiano Okalo, Brenda Apili, Viola Nalwoga, Samson Udho

**Affiliations:** 1 Department of Psychiatry, Faculty, of Health Sciences, Lira University, Lira, Uganda; 2 Department of Nursing and Midwifery, Faculty of Health Sciences, Lira University, Lira, Uganda; Universidad de Sevilla, SPAIN

## Abstract

**Background:**

Compassion fatigue is associated with negative consequences that undermine workplace performance. However, literature is scarce on compassion fatigue among nurses in the context of Uganda who are at higher risk of compassion fatigue owed to the nature of their occupation and the unfavorable work environment. We aimed to assess the prevalence and predictors of compassion fatigue among nurses in Northern Uganda.

**Methods:**

We conducted a cross-sectional survey among 395 randomly selected nurses from two referral and four general hospitals in Northern Uganda. Data was collected using a self-administered questionnaire. Data analysis consisted of descriptive statistics, cross-tabulations, and logistic regression at a 95% level of significance in SPSS version 25.

**Results:**

Of 395 nurses who took part in the study, 58.2% were female, 39.8% had a diploma, 47.1% were single, and 32.4% had worked for between 11–15 years. Close to 50% of the nurses experienced compassion fatigue. The predictors of compassion fatigue among the participants were; workplace bullying (AOR: 3.83; 95% CI: 1.99–7.36; *p =* <0.001) career enhancement opportunities (AOR: 9.16; 95% CI: 2.32–36.22; *p* = 0.002; and remuneration (AOR: 7.30; 95% CI: 2.73–19.49; *p =* <0.001).

**Conclusion:**

More than 3 in 4 nurses in Northern Uganda experience compassion fatigue. The Ministry of Health together with other stakeholders should strive to increase career enhancement opportunities for nurses, improve nurses’ remuneration and improve the overall workplace environment to prevent compassion fatigue among nurses in the country.

## Introduction

More than half of health professionals in high-income countries suffer from compassion fatigue [1, 2] and there is growing evidence to show that it affects nurses compared to other health professionals [3]. This may be because nurses directly support patients empathetically as compared to their counterparts [4]. In sub-Saharan Africa and East Africa in particular, there is limited evidence on the wellbeing of the health workforce [5], rather, there is a pre-occupation with concerns of the health systems shortfalls, specifically staff absenteeism, medical malpractices, understaffing, staff turnover, the availability, quantity, and quality of health services, and the poor working conditions of the workforce [6]. Nurses, the biggest single group of health care professionals are challenged to deal with the complex demands of overstrained health systems. These demands can result in compassion fatigue [7].

Compassion fatigue is defined as a state of reduced capacity to care as a result of constant exposure to the suffering of patients and patients’ traumatic experiences [8]. According to Coetzee and Klopper [9], compassion fatigue refers to the fact that nurses use more empathy energy than they need to recover, and therefore, the power to recover is lost. Based on a conceptual model, the term professional quality of life is used to evaluate compassion fatigue which consists of both positive (compassion satisfaction) and negative (burnout or dissatisfaction) components [10]. Although both compassion fatigue and burnout are used interchangeably, they are different and motivated by different circumstances and can lead to common outcomes such as absenteeism. Burnout is a result of the workload and institutional stress while compassion fatigue is due to exposure to working with those suffering from the consequences of traumatic events [11]. Compassion fatigue may involve but go beyond burnout where nurses may be less empathetic with patients [12]. It is common among caring professions and typically comprises diminished compassion, hypervigilance, intrusion, and avoidance [13]. In studies conducted among nurses in Scotland, Ireland, Jordan and Poland indicated that 75%, 64%, 52.3%, and 38.9% respectively had symptoms of compassion fatigue [14–17]. Other studies show that the prevalence of compassion fatigue in nurses ranges from 21.6% to 44.8% depending on the area to which they are assigned such as emergency and ecology [1]. A systematic review involving 6533 nurses across six different countries showed that 24.48% of the respondents had high levels of compassion fatigue [18].

Compassion fatigue has negative ramifications on both individuals and organizations. To the individuals, it may lead to strained relationships, sleep disturbance, alcohol and drug abuse, depression, suicide, and generally poor quality of life [19, 20]. To the organization, studies show that compassion fatigue in nursing leads to reduced quality of care, less satisfaction resulting in lateness, absenteeism, and nurses quitting the organization and possibly the nursing profession [19, 21, 22]. A recent report in Uganda suggests that more than 80% of health professionals want to quit which is evidence suggestive of compassion fatigue [6, 23]. This compassion fatigue appears both a consequence of the underlying health system inefficiencies such as perceived low pay, delayed salaries, lack of accommodation, and limited supervision that worsened health workers’ absenteeism [23]. Therefore, it can be concluded that health care professional workforce compassion fatigue worsened the impact of the existing staff shortages, a large number of patients, moonlighting, and exacerbated poor quality of health in the country.

The prevalence of compassion fatigue has been linked majorly to personal factors like gender, and working experience [18, 24, 25]. Organizational risk factors linked to compassion fatigue include work overload, low remunerations, unsupportive peers, lack of control, and ineffective supervision [18]. Other factors include coping strategy, self-compassion, social support, and professional cognition [18, 24, 25]. Available data on nurses shows a link between compassion fatigue and work overload and years of working experience [26]. However, other studies show that nurses with more years of experience and work overload had less compassion fatigue [27, 28].

The susceptibility of compassion fatigue among nurses may be even higher in northern Uganda not only due to shortage of nurses and overstretched health systems but also historical factors. The region was affected by a protracted brutal civil war that claimed tens of thousands of lives and many others displaced [29]. The war described as the "worst forgotten humanitarian crisis in the world" lasted from1986 to 2006. The Juba peace truce arranged in 2005 brought a shift in humanitarian efforts to economic recovery and health rehabilitation. The significant response to such events relied greatly on nurses. Admittedly the war ended but its impact on the health sector and individuals is evident [30]. Therefore, nurses are still working with critical patients. Nurses experiencing compassion fatigue have difficulties making appropriate clinical decisions and potentially are at risk of harming patients. Subsequently, scholars agree that compassion fatigue is an occupational hazard that professionals need to address [31]. Moreover, this topic has not been sufficiently studied especially in Uganda. The current study assessed the prevalence and associated factors of compassion fatigue in nurses in northern Uganda.

## Methods

### Study design and setting

A cross-sectional method study design was employed in one survey covering nurses working in northern Uganda. The study was conducted in two referral and four general hospitals in northern Uganda between 21^st^ February and 30^th^ March 2021. The region experienced a two-decade-long insurgency caused by the Lord’s Resistance Army (LRA) rebels that claimed thousands of lives and left many people displaced and homeless. Subsequently, the healthcare system in the region is weak and in a state of recovery. This has created an unsuitable working environment for the health workforce particularly, the nurses who are tasked with looking after critically ill patients.

### Participants and sample size

The study included all nurses registered with the Uganda Nurses and Midwives Council (UNMC) from different health centers in Northern Uganda. Nurses who were busy and or attending to critically ill patients at the time of data collection were excluded from the study. The sample size was calculated using a single population proportion formula [32]. The z-score (z) was 1.96, the margin of error (e) was 0.05, and the prevalence (p) for the known population was 0.5 since the proportion of nurses with compassion fatigue in Uganda is not known. The calculated sample size was 427 plus a 10% allowance for nonresponse. Eligible participants were randomly enrolled in the study until the desired sample was realized.

### Data collection tool and procedures

We used a self-administered questionnaire to collect data from the nurses across the six health facilities. We used a validated revised Stamm’s ProQOL V-5 tool to assess compassion fatigue among the nurses (r = 0.82) [10]. ProQOL V-5 has 30 items measuring compassion fatigue, compassion satisfaction, and burnout but only ten items measuring compassion fatigue were analyzed (**S2 Appendix**). The ProQOL is currently in its fifth revision of the original tool called Compassion Fatigue Self-Test Survey tool developed by Figley in 1995 [33] on a 5-point Likert scale of 1 to 5. The instrument has undergone a rigorous psychometric assessment to improve sub-scale validity and reliability [10]. The compassion fatigue sub-scale has an alpha scale reliability of 0.75 and inter-scale correlations indicate 5% shared variance (r = -0.14; co- σ = 2%; n = 1187) [10]. Compassion fatigue in this study was binned as a dichotomy of “*yes*” and “*no*”. We also captured demographic and workplace contextual factors conceptualized to be associated with compassion fatigue. The completion of the questionnaire by the study participants lasted for 20 to 30 minutes.

### Data management and analysis

Data were analyzed using SPSS version 23.0. Every questionnaire was checked for completeness at the end of each interview. A data entry screen was created in SPSS version 23 with checks to ensure accuracy during entry. Data were scanned for out-of-range and missing values before commencing data analysis. Categorical variables were analyzed using descriptive statistics, cross-tabulation, and logistic regression at a 95% level of confidence. Variables with a *p*<0.05 at the binary logistic regression level were included in the multivariate logistic regression model. The model fitness was checked using the Hosmer-Lemeshow test and a backward conditional logistic regression method was used to determine variables that were independently associated with the outcome variable. The effects of the independent variables were expressed as odds ratio (OR) and associated 95% confidence intervals (CI). The outcome variable of the study was compassion fatigue transformed into a dichotomous variable of yes and no.

### Ethical considerations

This study was approved by the Makerere University School of Medicine Research and Ethics Committee Institutional Review Board (UG-REC-027). Participants in this study were recruited based on written informed consent (**S1 Appendix**) and confidentiality was maintained throughout the entire research protocol process. The study was anonymous and participants had the right to withdraw at any time.

## Results

Out of the 427 calculated sample size, only 409 responded to the questionnaire giving a response rate of 95.7%. However, 12 questionnaires were excluded due to missing information on compassion fatigue giving a total of 395 participants for the final analysis.

### Demographic characteristics of study participants (N = 395)

The results in Table 1 indicate that 58.2% (n = 230) were female, 39.8% (n = 157) had a diploma, 47.1% (n = 186) were single and 32.41% (n = 128) had worked for between 11–15 years.

**Table 1:** Demographic information of the study participants (N = 395).

Variables	Category	Frequency	Percent	*p*-value
**Gender**				0.130
	Male	165	41.77	
	Female	230	58.23	
**Education levels**				0.074
	Certificate	117	29.62	
	Diploma	157	39.75	
	Bachelors’	111	28.10	
	Masters	10	2.253	
**Marital status**				0.349
	Single	186	47.09	
	Married	152	38.48	
	Widow/ Separated	57	14.43	
**Working experience**				<0.001
	1–5 Years	100	25.32	
	6–10 Years	92	23.29	
	11–15 Years	128	32.41	
	>15 Years	75	18.99	

### Prevalence of compassion fatigue among nurses in Northern Uganda (N = 395)

Based on the ProQOL manual guidelines, 49.11% (n = 194) had high levels of compassion fatigue, 29.62% (n = 117) reported average levels of compassion fatigue and 21.27% (n = 84) indicated that they had low levels of compassion fatigue (Table 2).

**Table 2:** Level of compassion fatigue.

Levels of compassion fatigue	N	% (95% CI)
≤22 (Low)	84	21.27 (17.23–25.30)
23–41 (Average)	117	29.62 (25.11–34.12)
(≥42) (High)	194	49.11 (44.18–54.04)

### Workplace contextual factors associated with compassion fatigue among nurses in Northern Uganda (N = 395)

The majority of the nurses (56.2%) experienced workplace bullying, 55% were less satisfied with their career enhancement prospectus at their workplace while the majority (52%) were satisfied with their current remuneration (Table 3).

**Table 3:** Workplace contextual factors associated with compassion fatigue among nurses.

Variables	Category	Frequency	Percent	*p*-value
**Workplace Bullying**				<0.001
	Yes	222	56.2	
	No	173	43.8	
**Career Enhancement**				<0.001
	Satisfactory	178	45.1	
	Less Satisfactory	217	54.9	
**Remuneration**				<0.001
	Satisfactory	204	51.6	
	Less Satisfactory	191	48.4	
**Quitting**				<0.001
	Yes	195	44.8	
	No	200	55.2	

### Predictors of compassion fatigue among nurses in Northern Uganda

In the multivariate logistic regression model in Table 4, the factors that were independently associated with compassion fatigue among the study respondents were: workplace bullying (AOR: 3.83; 95% CI: 1.99–7.36; *p =* <0.001) career enhancement opportunities (AOR: 9.16; 95% CI: 2.32–36.22; *p* = 0.002; remuneration (AOR: 7.30; 95% CI: 2.73–19.49; *p =* <0.001).

**Table 4:** Multivariable logistic regression analysis of predictors of compassion fatigue among nurses in Northern Uganda (N = 395).

		Compassion fatigue				
Variable	Category	No	Yes	COR 95%CI	AOR 95% CI	*p*-value
**Workplace Bullying**						
	Yes	17(7.7)	205(92.3)	7.62(4.26–13.64)	3.83(1.99–7.36)	<0.001*
	No	67(38.7)	106(61.3)	1.00	1.00	
**Career Enhancement**						
	Satisfactory	8(4.5)	170(95.5)	11.45(5.35–24.54)	9.16(2.32–36.22)	0.002*
	Less Satisfactory	76(35.0)	141(65.0)	1.00	1.00	
**Remuneration**						
	Satisfactory	6(2.9)	198(97.1)	22.78(9.62–53.93)	7.30(2.73–19.49)	<0.001*
	Less Satisfactory	78(40.8)	113(59.2)	1.00	1.00	

*: Statistically significant variables at p<0.05; COR: Crude Odds Ratio; AOR: Adjusted Odds Ratio; 1.00: Reference category.

## Discussion

We examined the prevalence and contextual factors associated with compassion fatigue among nurses in northern Uganda. Findings in our study demonstrated that close to 50% had high levels of compassion fatigue. Our findings suggest that the nurses are exposed to traumatic materials of their patients experiencing distress. The presence of small acts of empathy that takes on the meaning and mark patients’ continuing memories of the overall experience of the health care received is enough to cause compassion fatigue. Additionally, the findings suggest that nurses may be lacking sufficient skills in coping with the traumatic experiences of their patients. The insufficient competencies may be compounded by poor working conditions and an unsupportive environment [34]. The adverse effects of compassion fatigue impend the provision of empathetic care to patients [35]. Overall, the results confirm results from previous studies which showed that working with patients who are in pain and witnessing traumatic labor impact the mental health of nurses [28, 35]. Compared to previous studies, our findings reported higher levels of compassion fatigue than 44.8% in Greece, 38.9% in Poland, and 24.2% in China [36–38]. However, our results were lower than 59%, 52%, 75%, and 64% reported by nurses in Portugal, America, Scotland, and Jordan respectively [14–17, 39, 40]. This incongruence may be attributed to differences in sample sizes, departments, cultures, regions, resources, and working environments in different countries.

Genders, marital status, and education were not significantly associated with compassion fatigue. These results are not surprising because previous studies reveal inconsistent findings. However, work experience was significantly associated with compassion fatigue. Our results are in tandem with previous research which shows no association between compassion fatigue and demographic factors [25, 39, 41, 42]. Other studies, however, reveal a statistically significant association between compassion fatigue and demographic factors [38, 43, 44]. According to Hill and Stephens [45], compassion fatigue lowered with increasing working experience. In a systematic review of 71 studies, an association between socio-demographic characteristics and compassion fatigue was inconsistent [46]. Therefore the association between compassion fatigue and demographic factors remains inconclusive.

Results indicate that nurses who experienced workplace bullying were more likely to experience compassion fatigue compared to their counterparts who did not experience workplace bullying. This result is not surprising because workplace bullying is more frequently reported among nurses than people in other professions [47] and has negative ramifications. This bullying usually takes numerous forms including job-related bullying, intimidation-related bullying, and personal bullying. The negative ramifications of workplace bullying include loss of confidence, reduced self-esteem, depression, and compassion fatigue [48] as reflected in our study. Our results are in line with the findings of Kim and colleagues [49] which showed that workplace bullying had a significant effect on compassion fatigue. Findings from Laeeque and colleagues’ [50] study on compassion fatigue in nurses in Pakistan revealed similar results.

We observed in the current study that nurses who expressed satisfaction with their career enhancement opportunities in their current workplace were nine times more likely to experience compassion fatigue compared to their colleagues who were less satisfied with their career enhancement prospects. This is a surprising result because it is a well-known fact that lack of career enhancement opportunities leads to work-related stress [51] which may precipitate compassion fatigue. However, research indicates that the factors causing compassion fatigue in nurses include patient care, lack of skills, lack of communication, feeling the responsibility for the deadly ill, lack of communication, lack of social support, and increased intensity of patients [52, 53]. Therefore, while little is known about the association between career enhancement opportunities and compassion fatigue, it is apparent that compassion fatigue is a feeling resulting from experiencing an event that includes trauma-related stress [54]. In contrast to our findings, Andriani et al., [41] found a significant association between compassion fatigue and pay satisfaction.

Nurses who reported that their current remuneration was satisfactory were seven times more likely to experience compassion fatigue compared to those who were less satisfied with their current pay. This was an unexpected result which nevertheless is understandable. First, compassion fatigue is an occupational hazard because simply being in nursing places one at risk [55] regardless of the remuneration. Second, compassion fatigue is a natural result of empathy. In the context of the current study, it seems that the nurses who felt that they were satisfied with their remunerations empathized with their patients, identified themselves with patients more easily, and were exposed to traumatic materials of the patients. According to Figely [54], the more the level of empathy the higher the risk for compassion fatigue. These nurses seem to experience compassion fatigue because of proximity to tragedy over time, they seem not to separate themselves from the source of work-related stress [56]. Third, these nurses seem to be overcommitted to their jobs and do not invest in their personal quality of life putting them at risk for compassion fatigue [57].

### Limitations of the study

The results of this study aimed at providing clarity regarding the prevalence and the predictive factors of compassion. However, we are unable to infer causality from the observed association due to the cross-sectional design used in the study. The study also used self-report questionnaires with the possibility of recall bias in the results. Additional research is needed to expand and clarify the present results of compassion fatigue among nurses in Uganda.

## Conclusion

More than 3 in 4 nurses in Northern Uganda experience compassion fatigue. Workplace bullying, career enhancement opportunities, remuneration, and working experience are predictors of compassion fatigue. The findings of this study give insights into what should be done to improve the workplace environment of nurses in Uganda. These results can be used by nursing leadership and other stakeholders to create an enabling environment for nurses to promote job satisfaction and avoid the brain drain of the skilled nursing workforce. More research is needed to understand how compassion fatigue is correlated with burnout and job satisfaction.

## Supporting information

S1 Appendix Consent form. (DOCX)

S2 Appendix Stamm’s ProQOL V-5. (DOCX)
