# Supplementary material for: Prevalence and contextual factors associated with compassion fatigue among nurses in northern Uganda
Source: PLoS One. 2021 Sep 27;16(9):e0257833. doi: 10.1371/journal.pone.0257833 (PMC8475982; doi:10.1371/journal.pone.0257833)
Supplement: S1 Appendix — (DOCX) [file pone.0257833.s001.docx]

**S1Appendex. Consent form**

**Title of the study:** Prevalence and contextual factors associated with compassion fatigue among nurses in northern Uganda

**Investigator(s):** Kabunga Amir Lucas Goodgame Anyayo, Okalo Ponsiano, Apili Brenda, Nalwoga Viola, Samson Udho

**Institution(s):** Lira University

**Introduction:** We are Lecurers of Lira Universty in Public Health and Psychitry. We are conducting a study on the ***Prevalence and contextual factors associated with compassion fatigue among nurses in northern Uganda***. This informed consent explains the study to you. After the study has been explained, any questions you may have are answered, and you have decided to participate in the study, you will be asked to sign a consent, which you will be given a copy to keep.

**Purpose:** To asses ***Prevalence and contextual factors associated with compassion fatigue among nurses in northern Uganda***

**Procedures:** Your participation in this study will involve filling in a questionnaire with help of the researcher. All nurses working in nurses are eligible to participate in the study.

**Who will participate in the study?**

You have been chosen to participate in this study because you are nurses working in nurses. The interview will last for approximately for 25-30 minutes.

**Risks/discomforts:** There is no foreseeable risk of harm or discomfort that will arise from your participation in this study. The only risk or discomfort will be the inconvenience in terms of time spent during the interview.

**Benefits:** Knowledge gained from the study will be to understand the the influence of HIV-related stigma on new infections among HIV-positive young women in a rural setting.

**Confidentiality:** Your identity will not be revealed to any one as we shall only use codes to identify participants. Information obtained will only be accessible by the research team. Soft copies of the data will be protected by password and hard copy files will be kept under lock and key. Confidential information will only be accessed by the principal investigator.

**Alternatives:** You do not have to participate in this study if you are not interested. You will not lose any benefit in case of no participation.

**Cost:** There will not be any additional cost incurred as a result of participating in this study. However, you will be provided with refreshments during the interview.

**Questions:** If you have any questions related to the study, or your rights as a research participant, you can contact the principal investigator, **Kabunga Amir** on telephone number 0777929576 or via email on [amirkabunga070@gmail.com](mailto:amirkabunga070@gmail.com).

**Statement of voluntariness:** Participation in the research study is voluntary and you may join on your own free will. You have a right to withdraw from the study at any time without penalty.

**Statement of consent**

........................................................................... Has described to me what is going to be done, the risks, the benefits involved and my rights as a participant in this study. I understand that my decision to participate in this study will not affect me in any way. In the use of this information, my identity will be concealed. I am aware that I may withdraw at any time. I understand that by signing this form, I do not waive any of my legal rights but merely indicate that I have been informed about the research study in which I am voluntarily agreeing to participate. A copy of this form will be provided to me.

Participant……………………. Signature/Thumbprint……………Date…….

Witness………………………. Signature……………………Date……………

Name of Interviewer……………………. Signature……………………Date………….
