# Supplementary material for: Prevalence and contextual factors associated with compassion fatigue among nurses in northern Uganda
Source: PLoS One. 2021 Sep 27;16(9):e0257833. doi: 10.1371/journal.pone.0257833 (PMC8475982; doi:10.1371/journal.pone.0257833)
Supplement: S2 Appendix — (DOCX) [file pone.0257833.s002.docx]

**S2Appendix. Stamm’s ProQOL V-5**

| **Items** | **Never** | **Rarely** | **Sometimes** | **Often** | **Very Often** |
| --- | --- | --- | --- | --- | --- |
| I am preoccupied with more than one person I help |  |  |  |  |  |
| I jump or am startled by unexpected sounds |  |  |  |  |  |
| I find it difficult to separate my personal life from my life as a care-giver |  |  |  |  |  |
| I think that I might have been affected by the traumatic stress of those I help |  |  |  |  |  |
| Because of my work, I have felt ‗on edge‖ about various things. |  |  |  |  |  |
| I feel depressed because of the traumatic experiences of the people I help |  |  |  |  |  |
| I feel as though I am experiencing the trauma of someone I have helped |  |  |  |  |  |
| I avoid certain activities or situations because they remind me of frightening experiences of the clients I help |  |  |  |  |  |
| As a result of my work, I have intrusive, frightening thoughts |  |  |  |  |  |
| I can‘t recall important parts of my work with trauma victims |  |  |  |  |  |
